# In-situ Attenuation Corrections for Radiation Force Measurements of High Frequency Ultrasound With a Conical Target

**DOI:** 10.6028/jres.111.034

**Published:** 2006-12-01

**Authors:** Steven E. Fick, Dorea Ruggles

**Affiliations:** National Institute of Standards and Technology, Gaithersburg, MD 20899; School of Architecture, Program in Architectural Acoustics, Rensselaer Polytechnic Institute, Troy, NY 12180

**Keywords:** attenuation correction, conical target, in-situ attenuation, power measurement, radiation force balance, radiation pressure, ultrasonic power, ultrasound power

## Abstract

Radiation force balance (RFB) measurements of time-averaged, spatially-integrated ultrasound power transmitted into a reflectionless water load are based on measurements of the power received by the RFB target. When conical targets are used to intercept the output of collimated, circularly symmetric ultrasound sources operating at frequencies above a few megahertz, the correction for *in-situ* attenuation is significant, and differs significantly from predictions for idealized circumstances. Empirical attenuation correction factors for a 45° (half-angle) absorptive conical RFB target have been determined for 24 frequencies covering the 5 MHz to 30 MHz range. They agree well with previously unpublished attenuation calibration factors determined in 1994 for a similar target.

## 1. Introduction

Radiation pressure [1–13] has been employed in a wide variety [14–27] of ultrasound power meter designs. In these instruments, an appropriately constructed target properly aligned in a steady-state underwater ultrasound field is subjected to a radiation force *F* given by

$$F=e_{T}W/c$$ (1)

where *e_T_* is a correction factor determined by various properties of the target, *W* is the time-averaged spatially integrated power intercepted by the target, and *c* is the speed of sound in the water. Time-averaging of the ultrasound occurs because, under practical circumstances, the inertia of the target causes it to effectively integrate pulses into the corresponding steady-state force. Spatial integration is a consequence of the extended geometry of all targets and is optimized in practical instruments by using targets larger in cross section than the incident beam.

One such instrument was designed and custom built at the National Bureau of Standards (NBS) to be a primary standard for ultrasound power, and served this purpose for the 24 years (1977–2001) that NBS/NIST ultrasound power measurement services were available to the public. The features which distinguish its design from all other known RFB designs, and the evolution of the means used to implement these features, have been described extensively elsewhere [23,26–27]. This article presents a detailed description of the method used since 1994 to correct for the attenuation of ultrasound within the NIST RFB in the configuration used to measure the output of ultrasound power transfer standards [29–30]. Empirical attenuation correction factors determined in 2005 are presented for 24 frequencies covering the 5 MHz to 30 MHz range, and are compared with previously unpublished attenuation calibration factors determined in 1994.

### 2.1 RFB Targets

Selection of the optimal shape and material for an RFB target depends on the usual variety of application-specific details. Combinations of constraints on accuracy, cost, propagation medium, ultrasound amplitude, frequency, spatial (scanned v. stationary), and temporal (continuous v. pulsed) characteristics have led to the use of flat, waffled, concave, and convex shapes, and to the use of both absorptive and reflective materials [14–27].

A useful feature of the conical target shape was identified by Borgnis [5], who found that for a conical target of half-angle *ϕ* centered in an unfocused ultrasound beam with propagation axis parallel to that of the cone,

$$e_{T}=1-\beta^{2}\cos2\phi$$ (2)

where *β* is the amplitude reflection coefficient of the target. From Eq. (2) it follows that *e_T_* can be set to unity for all values of *β* by the simple expedient of setting *ϕ* to 45°.

Results of experimental research begun in 1974 at NBS have confirmed that *e_T_* can be made to differ from unity by less than 0.1 % by setting *ϕ* to 45° for targets made of metal or absorptive rubber. Because targets of this type were the only ones used in the work described in this article, no further mention of *e_T_* will be made.

### 2.2 *In-situ* Attenuation with the 45° Conical Target

Most applications require that RFB measurements be used to determine the total power *W*_0_ radiated from the output port of a transducer, rather than the power *W* intercepted by the RFB target.

**Fig. 1:**
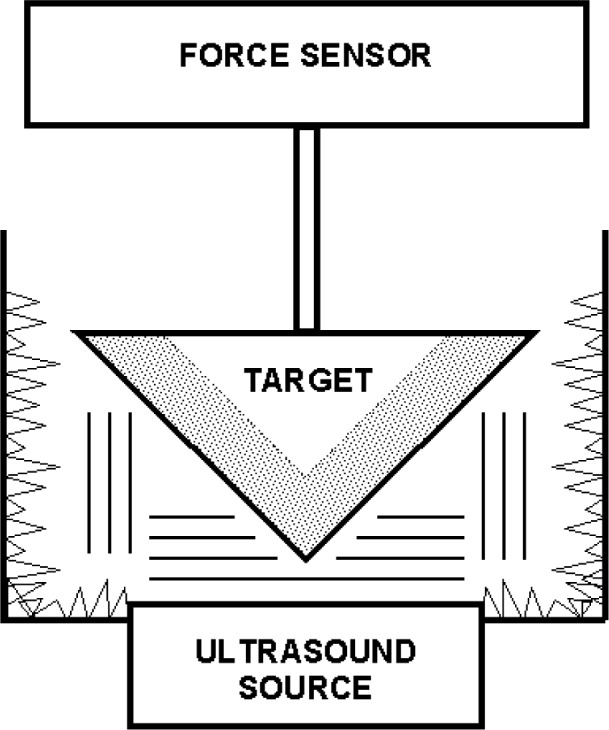
Simplified configuration of NIST RFB. Shading indicates silicone rubber conical shell of target.

For the RFB configuration depicted in Fig. 1, let *P* be the average ultrasound pressure in the plane which intersects the tip of the target and is perpendicular to the axis of the ultrasound beam. With a collimated, circularly symmetric ultrasound source of radius *R* located a distance *d* from this plane,

$$P=\frac{W_{0}e^{-ad}}{c\pi R^{2}}$$ (3)

where *α* is the empirically determined attenuation coefficient for the distilled water propagation medium, and *c* is the speed of sound in distilled water. An implicit condition for the validity of Eq. (3) is that *α* be independent of position in the ultrasound beam between the transducer and the target. This has long since been verified by experimental data, and is confirmed independently by recent data presented later in this article.

For the ultrasound sources used in this study, *P* is assumed to be uniform within the ultrasound beam. Because *ϕ* = 45°, the perpendicular distance from the apex plane to a point on an annulus on the target surface is identical to the radius of that annulus. Taking all annuli into account, the radiation force *F* induced on the target is given by

$$F=\int_{0}^{R}Pe^{-\alpha r}2\pi rdr$$ (4)

which reduces to

$$F=\frac{2\pi P}{\alpha^{2}}\left[1-(\alpha R+1)e^{\alpha R}\right]$$ (5)

Combining Eqs. (1), (3), and (5), we define attenuation correction factor *C* as

$$C=W_{0}/W=\frac{\alpha^{2}R^{2}}{2e^{-\alpha d}\left[1-e^{-\alpha R}(\alpha R+1)\right]}$$ (6)

## 3. Experimental Procedure

*In-situ* attenuation was determined empirically by measuring the output of a NIST Standard Ultrasound Source (SUS) [29–30] for 11 values of *d* covering the range 1 mm to 11 mm in 1 mm increments indicated with approximately 0.1 mm resolution. The target for all tests was a 45° half-angle conical shell of silicone rubber, 10 mm thick (measured axially) and 52 mm in diameter, surrounding an inner cone of rigid polyurethane foam. An ultrasound ray arriving parallel to the axis of the cone is reflected only slightly at the water-rubber interface because the rubber offers a good acoustical impedance match to that of water. In passing through the rubber, the ultrasound loses most of its energy to absorption. Near total reflection occurs at the interface between the rubber and the polyurethane foam, and the reflected ultrasound is further attenuated as it travels outward radially through the rubber layer. With most of the incident ultrasound absorbed, the remainder is reflected radially outward toward the cylindrical wall of the test tank. This wall is lined with irregularly oriented wedges made of AptFlex F36^1^ acoustic absorbing rubber. These wedges are approximately 20 mm by 25 mm at the base, approximately 35 mm long, and extend radially inward from the cylindrical wall.

The SUS transducer was installed in a customized test tank fitting which allows direct coupling to the water in the test tank with no intervening membrane. The transducer was positioned so that its output plane was several millimeters above the bottom surface of the tank. This arrangement ensures that any radiated edge waves will impinge on the absorptive wedges instead of being scattered from the bottom surface of the tank.

For all tests, the radio frequency drive voltage was adjusted to obtain 5.000 volts at the SUS dc monitor output [30]. This corresponds to output power levels equivalent to roughly 40 % of the rated SUS maximum power output [29–30]. Variations in drive voltage greater than 0.02 % were prevented by custom-built automatic-level-control circuitry [26]. Because the radiation conductance of the SUS transducer varies with frequency, the transducer power output varied from 348 mW to 455 mW for the 4.8 MHz to 29.2 MHz test frequency range. The test frequencies corresponded to odd harmonics of the approximately 0.5 MHz fundamental resonance frequency of the SUS transducer, and covered the test frequency range in increments of approximately 1 MHz.

For each of 11 values of liftoff distance *d*, the water temperature *T* was measured and recorded and the SUS dc monitor voltage was verified. Then 5 measurements of the intercepted power *W* were made. Each measurement was made by manually adjusting the magnitude and phase of a nulling signal to achieve an adequate null as indicated by an oscilloscope and galvanometer. The nulling voltage was then recorded in a spreadsheet which used the test temperature and an RFB scale factor to calculate the corresponding value of *W*.

System stability was monitored for each set of 5 measurements by calculating σ/μ, the relative standard deviation of the mean. The lower limit of this parameter for NIST RFB measurements made under typical circumstances [27] is 0.8 %. By this measure, system performance exceeded expectation during the course of the work reported in this article, as the largest value of σ/μ for any data set was 0.31 %.

The time of day was logged at the beginning of each set of measurements. The average time required to make each of 1320 measurements was 29 seconds. Results for a typical test are shown in Table 1.

## 4. Data Analysis

**Fig. 2:**
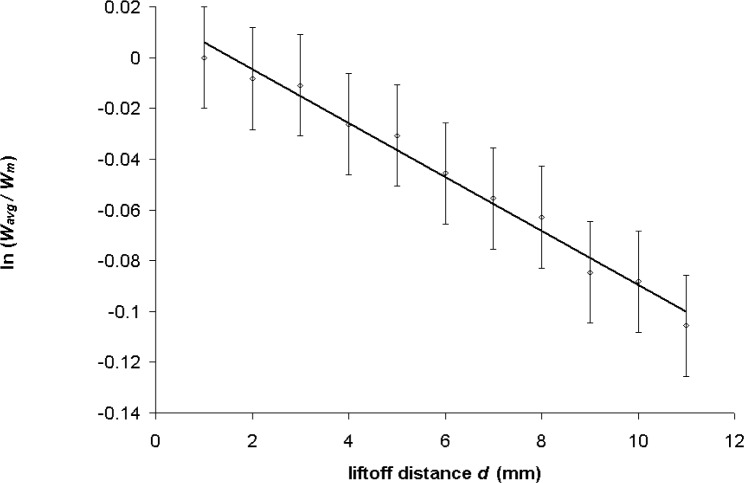
Variation of log-normalized power with liftoff distance, *f*=17.495 MHz.

For each set of 11 power measurements for a particular ultrasound frequency, the *in-situ* attenuation coefficient of water was calculated from the slope of a linear regression fit of ln(*W_avg_*/*W_m_*) to *d*, where *W_m_* is the average power intercepted by the RFB target with *d* at its minimum value. Results for the data of Table 1 are plotted in Fig. 2. The linear least-squares regression trendline in the figure lies within the bounds of error bars that reflect the 2 % best-case combined relative expanded power measurement uncertainty [27].

The results for all test frequencies are shown in Table 2. Attenuation coefficient *α*, in nepers per meter, is the slope of the trendline of the linear least-squares regression fit of ln(*W_avg_*/*W_m_*) to *d*, with *d* expressed in meters. The parameter *R* is the Pearson product-moment correlation coefficient [28]. To make it easier to see how closely *R* approaches unity in all cases, the parameter 1-*R* is tabulated instead.

The variation of 1-*R* with *f* in Table 2 indicates that the goodness of fit does not vary significantly over the range of *f*. The high values of *R* for all test frequencies confirm that *α* was constant throughout the volume of water defined by the ultrasound beam between the transducer and the surface of the target. Because this volume changed significantly as liftoff distance *d* was increased by 10 mm, the correlation indicated by *R* would have been compromised if *α* had changed significantly within this volume.

### 4.1 Long-term Reproducibility

The tests just described were conducted in June and July of 2005. To explore the effects of slight variations in system characteristics, we next examine the results of similar tests conducted in September and October of 1994 using a target which was identical except that its inner cone was constructed of polystyrene foam, rather than the polyurethane foam used in the target tested in 2005. The other systematic differences applicable in 1994 were the use of a test tank equipped with wedges made of organic rubber, instead of AptFlex F36, and the use of four SUS transducers, rather than one. The results of these tests are presented in Table 3.

A cursory inspection suffices to establish that the variation of 1-*R* with *f* in 1994 was about the same as it was in 2005. The calculated average values of 1-*R*, 0.0034 for the 1994 data and 0.0027 for the 2005 data, are insignificantly different.

### 4.2 Frequency Dependence

With *α* now available for a number of ultrasound frequencies determined by transducer resonances, we next consider interpolation schemes for calculating *α* for arbitrary frequencies within the 5 MHz to 30 MHz range. Methods involving fitting *α* to *f* ^2^ are attractive because the calculated slope is directly comparable with the published attenuation coefficient for distilled water [31]. Least-squares linear regression results for all data in Tables 2 and 3 are shown in Table 4.

While only slightly different from each other, the two slopes are substantially smaller than 0.0472 Np m^–1^ (MHz)^–2^, the attenuation coefficient for water at 22° C derived from values reported by Bacon and Jarvis [31] for distilled water and ideal (free-field plane wave) conditions. This discrepancy is attributed to the streaming wave motion that occurs when some of the ultrasound energy lost to attenuation is translated into momentum, in effect inducing a radiation force on the water itself. Because the NIST RFB uses audio-frequency pulse modulation of the ultrasound being measured, the ultrasound energy lost to streaming takes the form of water waves from which the target cannot be isolated because isolating membranes with acceptable ultrasound transmission characteristics inevitably would offer negligible resistance to the transmission of audio frequency water waves. With the attenuation in water virtually nil for audio frequencies, the amplitude of these water waves is undiminished as they travel to the surface of the target to induce forces which add to those induced by the ultrasound itself, reducing the apparent attenuation of the ultrasound from the source [26].

### 4.3 Attenuation Correction Factor Results

We begin by examining the accuracy of the least-squares-fit interpolation method for determining values of *α* for arbitrary frequencies. Hereinafter, all values of *C* will be calculated for liftoff distance *d* =1 mm, the value typical of NIST RFB operations, and for *R*=7.938 mm, the radius of the NIST SUS transducer.

For each of the 24 frequencies of the tests conducted in 2005, Fig. 3 shows values of *C* based on two values of *α*, one determined from the test results for that frequency, and the other determined by interpolation using the regression results of Table 4. For clarity, the bounds of the applicable 2 % combined relative expanded power measurement uncertainty [27] are shown as continuous curves rather than error bars.

The differences in *C* for every frequency are well within the bounds of even the best-case power measurement uncertainty, and show that the uncertainty due to the use of this interpolation method contributes only slightly to the overall uncertainty.

**Fig. 4:**
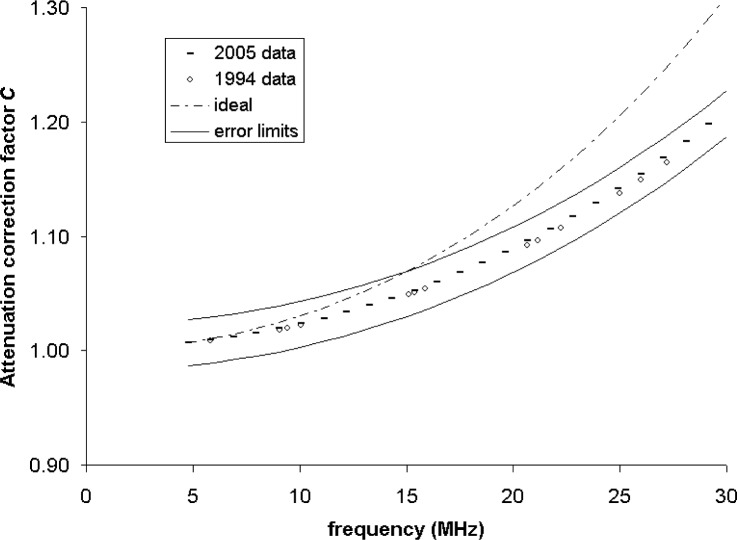
Attenuation correction coefficients for ideal and empirical values of α.

We next examine the consistency of attenuation correction coefficients determined from the two data sets 11 years different in age. Figure 4 shows values of *C* based on values of *α* from all test results reported herein for 1994 and 2005, and also for the attenuation coefficient for ideal conditions [31]. For all frequencies, differences in *C* from the 1994 and 2005 data sets are small compared to power measurement uncertainties. This agreement is taken to confirm that the procedures for determining *C* are fully adequate for the intended purpose.

It is also clear that values of *C* based on empirical and ideal values of *α* differ insignificantly for frequencies below 10 MHz, and substantially for frequencies above 15 MHz. To the extent that similar behavior applies to standard source output power measurements made by other RFBs equipped with conical targets, similar plots could assist the determination of the frequency range for which attenuation corrections are necessitated by constraints on overall uncertainty.

## 5. Conclusions

An expression has been derived for the *in-situ* attenuation correction factor *C* for a 45° conical target used to measure the output of a collimated, circularly symmetric ultrasound source.

*In-situ* attenuation *α* for distilled water was determined for 24 frequencies between 4.8 MHz and 29.2 MHz by least-squares linear regression analysis of measurements made in 2005 of ultrasound power versus source-target distance. This analysis also confirmed the validity of the assumption that *α* is independent of position in the volume of water defined by the ultrasound beam between the transducer and the NIST RFB target.

The combined accuracy of the derived expression and reproducibility of the performance of targets built to the NIST design was assessed by re-analysis of data from similar measurements made in 1994 with a different target. For the two data sets spanning 11 years, the average values of *R* differed by 0.0007, and had an average value of 0.9970, indicating good accuracy and reproducibility.

The results of least-squares linear regression analysis of the frequency dependence of *α* were found to confirm the expected strong correlation between *α* and *f* ^2^, and therefore to validate an interpolation method for determining *α* for arbitrary frequencies between 4.8 MHz and 29.2 MHz.

For this frequency range, the attenuation correction factor *C* was computed for both *in-situ* and ideal values of the attenuation of water. The difference between the two was found to be insignificant for frequencies below 10 MHz, and was found to exceed the best-case combined relative expanded power measurement uncertainty of NIST RFB power measurements [27] for frequencies greater than 15 MHz.

## Figures and Tables

**Fig. 3:**
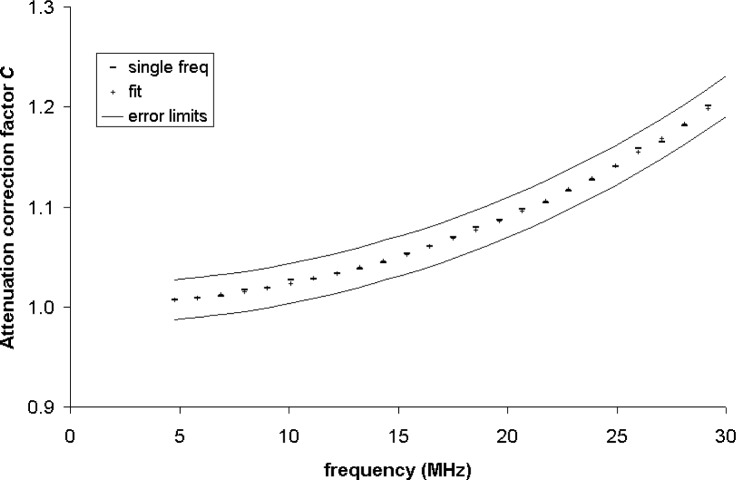
Attenuation correction coefficients for a from single-frequency 2005 data and least-squares fit of data for 24 frequencies

**Table 1:** Data for 17.495 MHz test conducted on 6 July 2005

Time EDT	*T* (°C)	liftoff *d* (mm)	*W1* (mW)	*W2* (mW)	*W3* (mW)	*W4* (mW)	*W5* (mW)	*W_avg_* (mW)
1341	21.7	1	396.5	397.0	396.8	396.7	397.2	396.8
1344	21.7	2	392.9	393.2	394.0	393.7	393.5	393.5
1349	21.7	3	392.8	393.5	393.4	391.6	391.3	392.5
1353	21.7	4	386.6	386.6	386.5	386.5	386.5	386.5
1355	21.7	5	384.9	385.0	384.7	384.7	384.7	384.8
1356	21.7	6	378.4	379.5	379.1	379.2	379.1	379.1
1358	21.7	7	375.4	375.3	375.4	375.4	375.6	375.4
1359	21.7	8	372.3	372.6	372.8	372.8	372.4	372.6
1401	21.7	9	364.7	364.7	364.6	364.2	364.6	364.6
1402	21.7	10	363.6	363.5	363.3	363.3	363.0	363.3
1404	21.7	11	356.8	357.2	356.4	357.2	357.2	357.0

**Table 2:** NIST RFB *in-situ* attenuation coefficients, from tests conducted in 2005

*f* (MHz)	*α* (Np/m)	1-*R*	*f* (MHz)	*α* (Np/m)	1-*R*
4.768	1.10	0.004	17.495	10.6	0.008
5.828	1.37	0.011	18.556	12.1	0.001
6.890	1.70	0.002	19.617	13.1	0.001
7.952	2.63	0.004	20.679	14.7	0.001
9.013	2.89	0.001	21.740	15.7	0.001
10.072	4.22	0.010	22.799	17.4	0.002
11.133	4.40	0.001	23.860	18.8	0.001
12.193	5.18	0.003	24.920	20.7	0.002
13.256	5.95	0.001	25.980	23.2	0.001
14.315	6.85	0.002	27.041	24.2	0.001
15.375	8.22	0.001	28.101	26.3	0.002
16.436	9.27	0.001	29.161	28.9	0.001

**Table 3:** NIST RFB *in-situ* attenuation coefficients, from tests conducted in 1994

*f* (MHz)	*α* (Np/m)	1-*R*	*f* (MHz)	*α* (Np/m)	1-*R*
5.828	1.32	0.017	20.679	13.6	0.003
9.060	2.40	0.006	21.140	15.3	0.001
9.447	2.76	0.003	22.248	15.0	0.002
10.072	3.60	0.001	24.980	21.2	0.001
15.100	7.41	0.007	25.980	21.6	0.001
15.375	8.29	0.002	27.205	24.9	0.001
15.860	8.77	0.001	30.220	28.9	0.002

**Table 4:** Least-squares fit results for NIST RFB *in-situ* attenuation coefficients. Slopes are expressed in Np m^–1^ (MHz)^–2^, intercepts in Np m^–1^

Date	α to *f*^2^ slope	α to *f*^2^ intercept	*R*
2005	0.0331	0.338	1.000
1994	0.0322	0.207	0.998

## Footnotes

1 Certain commercial equipment, instruments, or materials are identified in this paper to foster understanding. Such identification does not imply recommendation or endorsement by the National Institute of Standards and Technology, nor does it imply that the materials or equipment identified are necessarily the best available for the purpose.

## References

[1] Rayleigh L (1905). On the momentum and pressure of gaseous vibrations and on the connection with the virial theorem. Phil Mag 10 Series.

[2] Brillouin L (1925). Sur la tensions de radiation. Ann Phys.

[3] Biquard P (1932). Les ondes ultra-sonores. Rev Acoust.

[4] Bopp F (1940). Energetische Betrachtungen zum Schallstrahlungsdruck. Ann Phys.

[5] Borgnis FE (1952). Acoustic radiation pressure of plane-compressional waves at oblique incidence. J Acoust Soc Amer.

[6] Westervelt PJ (1957). Acoustic radiation pressure. J Acoust Soc Am.

[7] Kanevskii IN (1961). Steady forces arising in a sound field. Soviet Physics Acoustics.

[8] Rooney JA, Nyborg WL (1972). Acoustic radiation pressure in a traveling plane wave. Am J Phys.

[9] Livett AJ, Emery EW, Leeman S (1981). Acoustic radiation pressure. J Sound Vibration.

[10] Chu B-T, Apfel RE (1982). Acoustic radiation pressure produced by a beam of sound. J Acoust Soc Am.

[11] Beissner K, Makarov SN (1995). Acoustic energy quantities and radiation force in higher approximation. J Acoust Soc Am.

[12] Beissner K (1996). Acoustic energy quantities and radiation force in higher approximation. II. Boundary conditions of the Earnshaw-Riemann solution. J Acoust Soc Am.

[13] Beissner K (1998). The acoustic radiation force in lossless fluids in Eulerian and Lagrangian coordinates. J Acoust Soc Am.

[14] Sivian LJ (1928). A modification of the Rayleigh disk method for measuring sound-intensities. Phil Mag 7, Series 5.

[15] Barone A, Nuovo M (1951). Misuratore di intensita` ultraonora. Riceria Scientifica.

[16] McNamara FL, Beyer RT (1953). A variation of the radiation pressure method of measuring sound absorption in liquids. J Acoust Soc Am.

[17] Mokhtar M, Youssef H (1956). Sensitive electrodynamic balance for measurement of absorption of ultrasonic waves in liquids. J Acoust Soc Am.

[18] Wells PNT, Bullen MA, Freundlich HF (1964). Milliwatt ultrasonic radiometry. Ultrasonics.

[19] Kossoff G (1965). Balance technique for the measurement of very low ultrasonic power outputs. J Acoust Soc Am.

[20] Wemlen A (1968). A milliwatt ultrasonic servo-controlled balance. Med Biol Eng.

[21] Rooney JA (1973). Determination of acoustic power outputs in the microwatt-milliwatt range. Ultrasound Med Biol.

[22] Farmery MJ, Whittingham TA (1978). A portable radiation force balance for use with diagnostic ultrasound equipment. Ultrasound Med Biol.

[23] Greenspan M, Breckenridge FR, Tschiegg CE (1978). Ultrasonic transducer power output by modulated radiation pressure (with details). J Acoust Soc Am.

[24] Engan H (1982). An ultrasound power meter. J Acoust Soc Am.

[25] Beissner K, Ziskin M, Lewin P (1992). Radiation force and force balances. Ultrasonic Exposimetry.

[26] Fick SE, Breckenridge FR (1996). Ultrasonic Power Output Measurement by Pulsed Radiation Pressure. J Res Natl Inst Stand Technol.

[27] Fick SE (1999). Ultrasound Power Measurement by Pulsed Radiation Pressure. Metrologia.

[b28-v111.n06.a04] 28Microsoft Excel 97 SR-2, Help Contents and Index, RSQ worksheet function

[29] Fick SE, Breckenridge FR, Tschiegg CE, Eitzen DG (1984). An Ultrasonic Absolute Power Transfer Standard. J Res Natl Bur Stand.

[30] Fick SE, Ziskin M, Lewin P (1992). The NIST Power Reference Source. Ultrasonic Exposimetry.

[31] Bacon DR, Jarvis DR The speed and attenuation of sound, in Kaye and Laby Online Tables of Physical and chemical Constants.

